# Effectiveness and neural mechanisms associated with tDCS delivered to premotor cortex in stroke rehabilitation: study protocol for a randomized controlled trial

**DOI:** 10.1186/1745-6215-14-331

**Published:** 2013-10-12

**Authors:** Ela B Plow, David A Cunningham, Erik Beall, Stephen Jones, Alexandria Wyant, Corin Bonnett, Guang H Yue, Mark Lowe, Xiao-Feng Wang, Ken Sakaie, Andre Machado

**Affiliations:** 1 Department of Biomedical Engineering, Lerner Research Instt., Cleveland Clinic, 9500 Euclid Avenue, ND20, Cleveland, OH, 44195, USA; 2 Department of Physical Medicine & Rehab, Neurological Instt., Cleveland Clinic, Cleveland, OH, 44195, USA; 3 Imaging Institute, Cleveland Clinic, 9500 Euclid Avenue, U15, Cleveland, OH, 44195, USA; 4 Center for Neurological Restoration, Neurosurgery, Neurological Instt., Cleveland Clinic, 9500 Euclid Avenue, S31, Cleveland, OH, 44195, USA; 5 Human Performance & Engineering Laboratory, Kessler Foundation Research Center, 1199 Pleasant Valley Way, West Orange, NJ, 07052, USA; 6 Quantitative Health Sciences, Lerner Research Instt., Cleveland Clinic, 9500 Euclid Avenue, JJN3-01, Cleveland, OH, 44195, USA

**Keywords:** Stroke, Rehabilitation, Brain stimulation, Motor cortex (M1), Premotor cortex (PMC), Diffusion tensor imaging (DTI), Functional magnetic resonance imaging (fMRI), Resting state functional magnetic resonance imaging (rs-fMRI), Transcranial magnetic stimulation (TMS), Transcranial direct current stimulation (tDCS)

## Abstract

**Background:**

More than 60% of stroke survivors experience residual deficits of the paretic upper limb/hand. Standard rehabilitation generates modest gains. Stimulation delivered to the surviving Primary Motor Cortex in the stroke-affected hemisphere has been considered a promising adjunct. However, recent trials challenge its advantage. We discuss our pilot clinical trial that aims to address factors implicated in divergent success of the approach. We assess safety, feasibility and efficacy of targeting an alternate locus during rehabilitation- the premotor cortex. In anticipating variance across patients, we measure neural markers differentiating response from non-response.

**Methods/Design:**

In a randomized, sham-controlled, double-blinded pilot clinical study, patients with chronic stroke (n = 20) are assigned to receive transcranial direct current stimulation delivered to the premotor cortex or sham during rehabilitation of the paretic arm/hand. Patients receive the designated intervention for 30 min, twice a day for 3 days a week for 5 weeks. We assess hand function and patients’ reports of use of paretic hand. A general linear mixed methods model will analyze changes from pre- to post-intervention. Responders and non-responders will be compared upon baseline level of function, and neural substrates, including function and integrity of output tracts, bi-hemispheric balance, and lesion profile. Incidence of adverse events will be compared using Fisher’s Exact test, while rigor of blinding will be assessed with Chi-square analysis to ascertain feasibility.

**Discussion:**

Variable success of cortical stimulation in rehabilitation can be related to gaps in theoretical basis and clinical investigation. Given that most patients with severe deficits have damage to the primary motor cortex or its output pathways, it would be futile to target stimulation to this site. We suggest targeting premotor cortex because it contributes substantially to descending output, a role that is amplified with greater damage to the motor cortex. With regards to clinical investigation, paired cortical stimulation in rehabilitation has been compared to rehabilitation alone in unblinded trials or to unconvincing sham conditions. Transcranial direct current stimulation, a noninvasive technique of brain stimulation, which offers a more effective placebo and has a favorable safety-feasibility profile, may improve scientific rigor. Neural markers of response would help inform patient selection for future clinical trials so we can address limitations of recent negative studies.

**Trial registration:**

NCT01539096

## Background

### Arm/hand deficits post-stroke: evidence regarding adjunctive cortical stimulation

Stroke is the leading cause of long-term disability in adults [1]. More than 60% of survivors experience residual deficits of the paretic upper limb [2,3], where standard rehabilitation generates only modest gains [4]. A promising new approach involves delivering adjunctive stimulation to the brain. Early studies show that electrical stimulation delivered to the surviving primary motor cortex (M1) in the stroke-affected hemisphere in conjunction with rehabilitation facilitates outcomes of the paretic extremity [5-14]. Translational models suggest the effect is synergistic and emerges from augmented activity within targeted, residual M1 [5-7]. Despite its preliminary success, recent clinical trials have failed to witness an advantage of the paired approach in comparison to rehabilitation delivered alone [15-19]. Gaps in the theoretical basis and variability of experimental design are implicated [4,19].

### Gaps in the theoretical basis and variability in experimental design

Studies have invariably targeted M1 in the stroke-affected hemisphere [8-10,15]. Given that M1 and its output pathways are spared only in few patients with focal lesions [20,21], targeting M1 may be ineffective across most. It is thus important to deviate from the classical strategy of stimulating M1 while still realizing that effectiveness may vary depending upon who is enrolled. Well recovered patients demonstrate promise of additive stimulation [14,22], but disappointing results are observed in patients with severe impairments [16,18]. The obvious theoretical questions remain - are the effects of adjuvant cortical stimulation favorable for some versus others, and why?

The clinical utility of paired stimulation in rehabilitation is also marred by variability in design. The paired approach has been compared to either rehabilitation alone in unblinded trials [8-10] or to unconvincing sham stimulation [16,23] or to no control conditions at all [24,25]. Approaches of stimulation are extremely variable too, with little information about why one method may have greater utility than another. Invasive techniques (intracranial stimulation of residual M1) carry serious postsurgical risks [8], but even noninvasive methods (delivered from over the scalp and skull), such as repetitive transcranial magnetic stimulation, rTMS, may induce acute seizures [26]. Plus, rTMS is non-portable, and impractical to apply in chronic rehabilitation [27], unlike invasive stimulation [8-10]. Modes of stimulation are distinct too, with some facilitating activity of stroke-affected hemisphere, and others downregulating that of the unaffected [28].

Rehabilitation strategies are inconsistent; some groups employ laboratory-based training paradigms [13,14,22,29], whereas others use standard [8-10,16,17,23-25,30] or newer methods of rehabilitation [12,18,31]. It is critical to understand that the type of training that is combined with stimulation determines how generalizable the benefits would be. Improvements are specific for tasks that are strategically paired with stimulation [7,29,32], thus, choosing a rehabilitative intervention/paradigm that is applicable across most activities of daily living would indeed be more meaningful. Additionally, duration is important because singular sessions of paired stimulation and training tend to be positive [13,14] and hence, popular, but they may inflate and contort the clinical advantage that is truly offered by stimulation.

### Rationale for our approach

In addressing theoretical gaps in the present literature, we propose that premotor cortex (PMC) could serve as an alternate locus in the stroke-affected hemisphere. As a higher-order motor region, it contributes substantially (approximately 60%) and independently to descending motor tracts, plays a superior role in dexterity [33-35], and contributes more strongly with increasing damage to M1 [36,37]. Descending motor output from PMC has as strong a predictive power in prognosticating recovery as output from M1 [38,39]. However, in anticipating that effects of stimulation can vary across patients, we propose to assess pathologic and neural markers of individual recovery [4,19] to inform design of future trials.

Since variability in design, choice of stimulation and its mode, and type of rehabilitative strategy have impacted interpretation of the utility of paired stimulation in stroke rehabilitation, here, we suggest design-related modifications for upcoming trials. When selecting between methods of cortical stimulation, an alternative non-invasive technique may involve transcranial direct current stimulation (tDCS), which with passage of low-level current alters cortical excitability. tDCS may provide a better safety-feasibility ratio than intracranial and rTMS methods because it induces minimal risks, offers effective placebo and is low cost and easy to administer in patients with chronic conditions with concurrent therapies [14,18,22,40]. With respect to rehabilitation protocols, adopting a paradigm that is standardized yet customizable to patients’ goals and objectives would be more effective in promoting generalizability; an example is modified constraint-induced movement therapy (CIMT) [41], involving use of paretic limb in patient-specific tasks simulating activities of daily living during constraint of the unaffected limb. CIMT may also be effective as a paired rehabilitation technique because it is shown to facilitate activity in targeted, residual M1 and restore inter-hemispheric balance [41]; delivering adjunctive tDCS could then amplify its neural basis and effectiveness [12]. Long-term rather than short-term pairing with rehabilitation may carry greater utility. This is because the effects of short-term tDCS are transient [42], but longer staggered paradigms generate greater retention [42,43]. Incorporating extended treatment paradigms [16-18,24,44] would also be important for making decisions about long-term clinical relevance [13,14,22,29,45] of paired cortical stimulation and rehabilitation.

### Objectives and hypotheses

In line with our rationale, the objectives of the present pilot clinical study are to: 1) examine the safety, feasibility and effectiveness of CIMT paired with tDCS targeting PMC, versus CIMT delivered alone in alleviating impairments of the paretic hand in patients with chronic stroke; and 2) assess neural markers associated with response to paired paradigm versus CIMT alone.

We hypothesize that the paired approach involving stimulation of PMC in the stroke-affected hemisphere will be more effective than CIMT in improving hand function in chronic stroke. The paired approach will facilitate the individual’s specific neural substrates of recovery and will be safe to employ throughout the length of the study.

## Methods

### Study design

We use a pilot, randomized, sham-controlled, double-blind clinical study design. Twenty patients are being randomly assigned to PMC tDCS plus CIMT or sham tDCS plus CIMT groups. All outcomes are collected at pretest and at post-test after 5 weeks of intervention. At 3-month follow up, outcomes of hand function are collected to examine retention.

### Participants and recruitment

We are enrolling patients with chronic stroke with mild-to-moderate impairments in upper limb function [46,47], who fulfill the following prerequisites:

### Inclusion and exclusion criteria

The inclusion criteria are: age ≥21 years; chronic phase of recovery (>6 months) after first-ever stroke up to 20 years post-stroke; ability to extend fingers or thumb or wrist ≥10° and chief complaint of inadequate ability to use the paretic hand compared to its use prior to a single (ischemic or hemorrhagic) stroke.

The exclusionary criteria relate to contraindications of cortical stimulation [48,49] and imaging [36,37]. These include: cardiac pacemaker; metallic implant in the head; seizure disorder; medication-resistant epilepsy in a first-degree relative; use of any neuro- or psycho-active medications as published in recommendations [48]; history of fainting spells of undetermined etiology; pregnancy; implanted pumps/stimulators/shunts; any other neurological condition affecting sensorimotor systems, such as brain tumor, dementia, or substance abuse; severe cognitive deficits (mini-mental state exam score [50] <18); ongoing/recent (within 2 months) rehabilitation for upper limb.

### Recruitment

This is a single-center pilot clinical study. Patients with chronic stroke are being recruited from nine area hospitals within the Cleveland Clinic health system through outpatient and community-based stroke programs, referrals from providers, medical chart review, liaison with local support groups, and spontaneous demand through newspaper, radio and website advertisements.

### Ethics, consent, study organization and registration

The study is being conducted in agreement with the principles of the Declaration of Helsinki, and with the guidelines of Good Clinical Practice (GCP) of the International Conference on Harmonization of Technical Requirements for Registration of Pharmaceuticals for Human Use (ICH). The study protocol has been approved by the local and independent Ethics Committee of the Cleveland Clinic (Institutional Review Board). The study is also registered as a pilot clinical trial on clinicaltrials.gov (NCT01539096). Extramural funding for the present study is provided by the National Institutes of Health (NIH) for EP.

During the consent process, the investigator explains the benefits and risks of participation in the study and provides an informed consent form approved by the Institutional Review Board. To gauge whether subjects have sufficiently understood the study processes so as to make an informed decision, the investigator probes by asking open-ended questions about details contained in the consent form. Only patients who respond correctly and provide written informed consent by signing the consent document are enrolled in the study. Results will be published only with de-identified data.

### Initial screening

Patients are initially screened by telephone, followed by thorough chart review. The physical therapist, principal investigator and study coordinator evaluate subjects, who provide written informed consent in person, to ensure eligibility. Diagnosis and eligibility are confirmed at screening by a neurologist. Screening for neuroimaging and brain stimulation is repeated at each of these levels.

### Study assessments

Following confirmation of eligibility, the pretest is scheduled over 2 days, and involves evaluation of upper limb function and neural markers of change. Post-test, following 5 weeks of the assigned intervention, is similar to the pretest.

**Figure 1:**
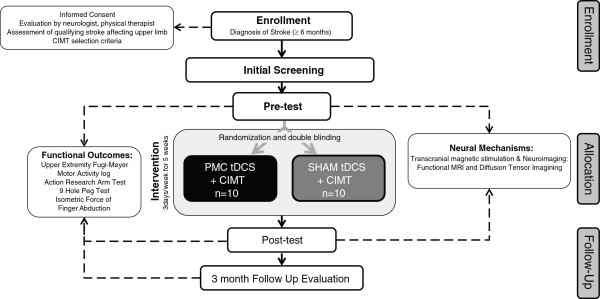
**Overview of study procedures.** CIMT, constraint-induced movement therapy; tDCS, transcranial direct current stimulation; MRI, magnetic resonance imaging.

Outcomes of hand function: the detailed timeline is presented in Figure 1. Our primary outcome of hand function is the upper extremity Fugl-Meyer (UEFM) assessment, which measures impairments of the upper limb/hand [51]. It is commonly used in studies pairing cortical stimulation with rehabilitation [8-10]. It includes 33 items that provide a gross indication of overall functional status of an individual. It is rated on an ordinal scale (0 to 2, maximum score 66) by an investigator [51].

Secondary outcomes of function include: 1) the motor activity log [47], which is a self-report assessment of the patient’s own perception of degree and quality of use of their affected hand in daily activities; 2) the Action Research arm test, which is a performance-oriented test that rates function across grasp, grip, pinch, and gross movement [52]; 3) the nine-hole peg test, which is a performance-oriented test of dexterity measuring time-to-place individual pegs consecutively into spaces and removing them as promptly as possible [53]; 4) isometric finger-abduction strength measured as force exerted during maximum isometric abduction; and 5) spasticity of finger and wrist flexors measured with the modified Ashworth scale [54], which ranges from 0 (no change in muscle tone upon quick stretch) to 4 (most severe spasticity, where the hand is rigid in flexion). All outcomes of hand function are repeated at a 3-month follow-up assessment.

### Markers of neural recovery

Neuroimaging and tests of cortical neurophysiology are being conducted at pretest and at post-test to identify predictors and correlates of response, compare which substrates are adaptive in one individual versus another, and discern the potential underlying reason for variance.

#### Structural neuroimaging

Predictors of response: diffusion tensor imaging defines the structural patency of white matter tracts [55]. Using this method, we evaluate the integrity of corticospinal tracts, which are considered most critical to dexterity [56]. We anticipate that PMC-based tracts will be more predictive of recovery with tDCS than those emerging from M1.

#### Functional neuroimaging

Mechanisms of response: functional neuroimaging is the most important assay to measure changes in the brain occurring in recovery in stroke. By measuring changes in perfusion or blood oxygenation during movement of the paretic hand, one can infer the association of activity of a region with its role in dexterity. Functional neuroimaging, such as functional MRI (fMRI), has demonstrated that damage due to stroke downregulates activity in stroke-affected versus intact hemispheres. As recovery occurs, the balance in activity (as seen using fMRI) between bilateral hemispheres, returns [37,41]. We are exploring whether delivering tDCS to PMC in the stroke-affected hemisphere helps restore this balance. Another form of functional neuroimaging, called resting-state fMRI [57], is critical in identifying how, over the course of stroke recovery, functional connectivity (fc) evolves between widespread networks [58]. We investigate whether fc is strengthened, particularly between PMC and M1, with application of tDCS in CIMT.

#### Neurophysiology

Substrates of response: transcranial magnetic stimulation (TMS) noninvasively measures cortical and corticospinal neurophysiology [59]. In the present pilot trial, TMS defines efficiency of conduction via residual corticospinal tracts [56], the structural integrity of which is outlined with diffusion tensor imaging. TMS also measures conduction between both hemispheres relayed via transcallosal pathways; this conduction is normally inhibitory, that is, one hemisphere exerts inhibition upon another, and that in return exerts counter-inhibition. In stroke, balance of such inhibition is disrupted. Whereas inhibition from the intact stroke-affected hemisphere is exaggerated, that in the opposite direction is diminished [60]. We explore whether tDCS delivered to PMC in the stroke-affected hemisphere improves the efficiency of corticospinal conduction and helps rebalance inhibition exerted between the intact and stroke-affected hemispheres.

### Randomization

We are using randomization blocks in sizes of four, where two patients in a block are assigned to PMC tDCS plus CIMT and the other two to the sham tDCS plus CIMT group. Such block randomization prevents the possibility that several patients in a row would get randomized to tDCS or sham tDCS groups in the early part of the study. The order of patients in blocks is random, generated using an online tool. Allocations are concealed in an opaque envelope and hidden in a locked cabinet with restricted access; they are opened by an investigator not involved in data collection or analysis before the first day of treatment.

### Interventions

#### CIMT

Once patients are randomly assigned to respective groups, patient-specific rehabilitation is delivered under supervision of the study principal investigator and physical therapist (EP) in an outpatient setting at the Cleveland Clinic main campus. All patients receive CIMT for 30 minutes, twice a day, 3 days a week for 5 weeks. It includes massed (intensive) functional exercises for the paretic upper limb guided by the principle of shaping [47], which involves a graded, regimented, feedback-driven approach to achieving impairment- and patient-specific goals. Tasks focus upon transfer to real-world activities, including activities like grooming, making a telephone call, et cetera. The protocol of CIMT discussed here differs slightly from the modified version of the program that is generally employed [46]. Instead of one 30-minute session, we deliver two 30-minute sessions; rather than 10 weeks, we deliver CIMT for 5 weeks, at the same frequency, that is, three times a week. We have incorporated this frequency and length to improve compliance, as patients are required to visit the outpatient clinic 15 times over 5 weeks rather than 30 times over 10 weeks. Patients’ use of the non-paretic upper limb is restrained by placing it in a mitt, which they are asked to maintain for 2 hrs every weekday during peak times of activity, instead of 5 hrs.

#### tDCS

The tDCS of anodal polarity is delivered to PMC in the stroke-affected hemisphere to potentially raise its activity [49]: it is delivered using a constant current battery-driven (9-V) stimulator (Soterix, NY, New York, USA) connected to conductive rubber electrodes (5 × 7 cm^2^) placed in saline-soaked sponges [61]. The anodal electrode is placed on the scalp site corresponding to PMC [34,61], guided by MRI-based stereotaxy. Specifically, the center of the anode is 3 cm anterior to the locus in the stroke-affected hemisphere that evokes the best and most consistent responses with TMS in a muscle of the paretic hand [61]. The reference (cathodal) electrode is placed above the contralateral orbit. Electrodes are secured using Velcro bands. Direct current is delivered at a dose of 1 mA during rehabilitation in patients in the tDCS plus CIMT group. For patients in the sham tDCS plus CIMT group, the 1 mA current is delivered transiently (30 to 60 seconds) at outset, and then slowly turned off after habituation. For all patients, the current is ramped up slowly at the onset of intervention to minimize excessive tingling and maintain blinding. This is a valid method for placebo as patients receiving tDCS become habituated to its sensation within a short time [62].

### Blinding

Patients and investigators assessing outcomes are blinded to the group assignment. Double blinding is intended to minimize bias that could emerge from participants’ perceptions of treatment and therapeutic confusion or observer bias within the investigative team about the benefits of the approach. To quantify the success of double blinding, we ask patients at the end of the study whether they believed they received tDCS or sham tDCS, or whether they did not have reason to believe one way or another. A similar questionnaire is completed with the investigators who assess outcomes.

### Safety

A report on side effects is completed at every visit. At every treatment visit, the report documents whether or not subjects experienced side effects related to tDCS, such as tingling, headache, itching, fatigue, pain and problems concentrating. On days of testing with TMS, the report also documents whether subjects experienced side effects related to TMS, such as seizure, loss of consciousness or hearing difficulties. Transient side effects are documented separately from serious events that are categorized as related or unrelated to interventions. Serious adverse events include those that may require inpatient hospitalization. Adverse event reporting follows guidelines set by the local Institutional Review Board. To ensure safety in case of seizure or any unrelated serious medical issues, provisions include an on-call medical response team and clinical research nursing support that work closely with the physician on the study (AM).

### Attrition

We are defining attrition as a lapse in treatment >1 week, inability to complete post-test or follow up, or exclusion or withdrawal in the case of a serious adverse event or development of a condition that is a contraindication to participation in the study.

### Sample size estimation

**Figure 2:**
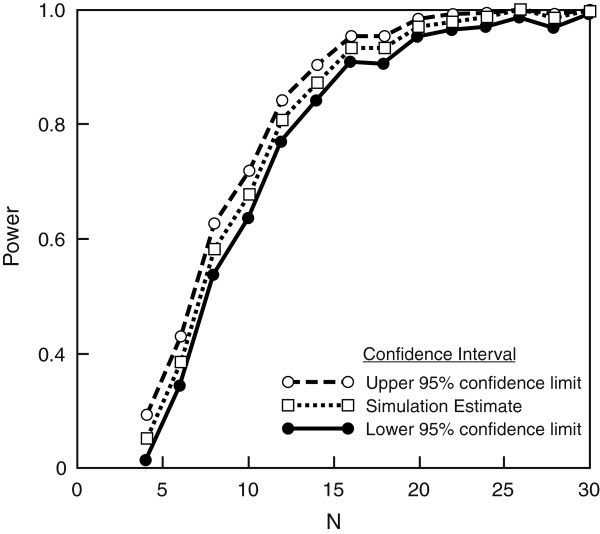
**Power analysis: power was determined by simulation.** Lower and upper lines define exactly the 95% CI for power, calculated based on the binomial distribution. N is the total sample size. The analysis was performed at a 0.05 significance level considering 2 × 2 factorial analysis of variance (ANOVA) (tDCS plus CIMT and sham tDCS plus CIMT from pretest to post-test). Power has been estimated here for an interaction term (A *C).

The sample size has been estimated based on previous studies that have paired invasive or noninvasive stimulation with rehabilitation [8,9,12,17,31]. Because UEFM has been most commonly investigated across the majority of studies, we used the effect sizes calculated across these studies to generate several permutations of sample size and the corresponding extent of change in the UEFM score (Figure 2) to estimate power. Using a method of simulations [63] where 500 simulations were performed, we estimated sample sizes that would generate significant differences upon the UEFM based on a mixed-effects model. From this method, we have found that a sample size of 10 per group would yield 83% power (95% CI 79.41 to 86.19%) to detect differences between groups (PMC tDCS plus CIMT sham tDCS plus CIMT group), 99.6% power to note differences across time (95% CI 98.56 to 99.95%) and 97% power (95% CI 95.35 to 99.46%) to observe a group X time interaction (Figure 2).

Although the estimated sample size appears small, it is commensurate with the pilot exploratory nature of our study. Pilot studies involving brain stimulation generally use smaller sample sizes compared to rehabilitation trials [28] because contraindications to brain stimulation/imaging are constraining. Still, we anticipate having adequate statistical power based on prior evidence where despite enrolling a total of 14 to 30 subjects, several studies report significant benefit of adjunctive stimulation [8,30,44]. Additionally, use of a repeated-measures design further provides greater power. Studying neural mechanisms with fMRI and tractography in limited sample sizes is challenging. Given the exploratory nature of our pilot study, we would be able to highlight elemental neural mechanisms of recovery. Previously, in a three-group, randomized controlled study design with a small sample of 20, we deciphered correlates of fMRI [37]. Recently too, Stagg *et al*. [64] show differences in fMRI activation across varying types of tDCS interventions in 11 patients with stroke. Without overemphasizing the importance of small pilot studies, we stress instead the significance of large-scale studies in generating clear evidence of efficacy. Our current pilot exploratory study is a step in that direction as it would offer estimates of effect size related to PMC tDCS and neural markers that can serve as entry criteria.

### Statistical and data analyses

Feasibility: analysis of feasibility includes investigating the rigor of experimental blinding and determining compliance with the 5-week outpatient rehabilitation program. A chi-square analysis will determine whether patients in one group versus another estimate their group assignment correctly. Investigators also complete a similar questionnaire at the end of the study. While patient responses will indicate the rigor of the blinding procedures, responses from investigators will ensure investigator equipoise, preventing observer bias. Attrition or missing data will be analyzed using a strategy known as multiple imputations [65], where each missing value is replaced with a set of plausible values that represent the uncertainty about the true value. The multiple imputed data sets are analyzed using standard procedures used for complete datasets and by combining results from these analyses. The method of multiple imputations does not attempt to estimate each missing value through simulated values, but rather represents a random sample of missing values. This process results in valid statistical inferences that properly reflect the uncertainty due to missing values.

Safety: the incidence of adverse events in each group will be computed as a proportion of individuals in each group who experienced side effects. We will be using the Fisher exact test to contrast the incidence of side effects between both groups.

Efficacy and markers of recovery: our primary endpoint of efficacy is the UEFM. Secondary outcomes include nine-hole peg test, Action Research arm test, motor activity log, finger abduction strength and the modified Ashworth scale of spasticity. A general linear mixed-model approach will be followed to analyze functional outcomes across three levels of time (pre, post, and follow-up). Significant two-way interaction, whenever present, will be explored using the Tukey honestly significant difference (HSD) test. Non-parametric tests will be used if the distribution is not normal.

To assess neural substrates of recovery, both groups will be compared for change in inter-hemispheric balance (fMRI), fc and transcallosal inhibition and corticospinal conduction (TMS) from pretest to post-test. To understand whether effects of interventions vary across patients and factors explaining such variance, an ideal method would have involved identifying clinical, pathologic and neural factors that would predict the change in UEFM. However, since the statistical power of the study may be inadequate to pursue this analysis, we will follow an alternative method. Patients who achieve ≥3.5 gain in the UEFM score (criterion of clinical improvement defined in a previous clinical trial of invasive cortical stimulation) [8] after 5 weeks of the intervention would be called responders. We will investigate whether the proportion of responders versus nonresponders includes a greater majority of patients from the tDCS group (Chi-square analysis). More importantly, we will compare clinical, pathologic and neural indicators between responders and nonresponders (independent samples *t*-test). These will include: baseline UEFM, location of disease (Oxford Stroke classification: total anterior circulation or partial anterior circulation or lacunar strokes) [66], residual function and integrity of corticospinal tracts on the stroke-affected side measured with TMS and diffusion tensor imaging respectively.

## Discussion

We have described the protocol of our ongoing pilot clinical study in chronic stroke, where we test safety, feasibility, efficacy and neural markers of upper limb rehabilitation combined with stimulation targeting the PMC in the affected hemisphere. Evidence on the value of paired cortical stimulation in rehabilitation is divided due to theoretical and design-related issues. Stimulating peri-lesional and affected M1 without knowing the individual substrates of recovery creates variability in response [4,19,67]. Differences in design, such as type of control, blinding and safety-feasibility trade off of varying types of stimulation have further added confusion to evidence of efficacy. Recent trials have also failed at the phase III level due to poor estimation of the magnitude of the placebo effect [19,68]. Therefore, to prepare for future trials, we propose targeting the PMC in the affected hemisphere with the rationale that it may serve as an alternative locus of recovery. We employ tDCS, which may be safer and feasible to apply online during long-term outpatient rehabilitation; based on its ability to provide a more effective placebo, it permits us to adopt double blinding and sham control, helping estimate the magnitude of placebo. Finally, we identify patient-specific indicators of recovery to stratify responders for design of future investigations.

Knowing markers of response versus non-response to brain stimulation in rehabilitation would not only harmonize evidence in the field, but will also help us select candidates for subsequent trials. Early clinical trials of invasive stimulation showed greater promise of adjuvant stimulation [8-10] than recent phase III [15,19], potentially since patients in earlier trials possessed functioning corticospinal pathways [9] unlike only a few in phase III [19]. Similarly, rehabilitative outcomes are invariably successful for well-recovered patients [14,22], unlike for those with moderate/severe paresis [16,18,31]. Subcortical lesions respond better [13,29] than massive infarcts of total anterior circulation [69]. If we find that responsive patients show distinctive characteristics, such as patent and functioning white matter tracts et cetera, then these would serve as entry criteria for the future. Unfortunately it may also mean that stimulation may be more fruitful only for a limited few, but this information would optimize resource allocation. For patients showing limited to no response, we would identify whether functioning of alternate areas (as identified on fMRI) and patency of tracts from other regions (from tractography) may hold greater meaning. A patient-guided approach to utilizing such residual resources will then be created, employing alternate rehabilitation methods and/or stimulating patient-specific loci.

Knowing mechanisms of long-term pairing can help address why chronic effects are not as robust. Even though singular sessions or short-term paradigms show benefit [11,13,16,22,29,30], trials incorporating long-term training have failed to witness an advantage [15-19]. Divergent success across short- and long-term studies may also be an effect of dosage. Stimulation may have an initial accelerative effect [25,32] manifested in the short term, but over the long term, the effect of rehabilitation itself may become robust [37,70], diminishing the comparative effect size. [15-19]. Confounding from individual variance may also factor in; if stimulation in rehabilitation were truly effective, its differences from rehabilitation would evolve over time; on the flip side, if all gains were solely related to variance across individuals, then a long-term study would show no difference, and short-term studies would be easily confounded. Short-term designs also have the added advantage of higher treatment fidelity; standardized laboratory-based paradigms can be delivered in short-term approaches more easily [13,14,22,29,45], whereas longer protocols of clinical rehabilitation suffer from variance in treatment delivery [8-10,16,17,23-25,30,44]. However, exploring stimulation delivered with long-duration rehabilitation still carries greater clinical utility, despite challenges to adherence and fidelity, because short-term paradigms, though invariably positive, do not correlate with long-term outcomes [13,14,22,23,29-31,45].

Use of chronic paradigms offers another important advantage, namely, retention. Besides the primary endpoint, we have built a delayed follow up. Initial clinical trials note that groups receiving cortical stimulation retain benefits whereas the ones receiving rehabilitation alone show a dip in performance after the end of training [8,9]. These delayed benefits, maintained post-stimulation, may be indicative of a neuro-protective effect [6] that we may be able to infer by completing our 3-month follow up.

Besides duration and retention, we suggest other potential advancements that could help improve the rigor of study design in the field. Creating a sham-controlled, double-blinded approach remains ethically and technically challenging with invasive stimulation [8-10], and sham conditions created with noninvasive rTMS have not been strongly convincing either [24,25]. Using tDCS may offer a unique opportunity to create a more valid sham for effective blinding [62] and provide a better estimation of the placebo effect. Caveats remain to be addressed, however. Is it possible that what is gained in safety-feasibility with tDCS is lost in effectiveness, when relating to more focal methods such as rTMS and invasive cortical stimulation [4].

Overall, the strengths of our pilot protocol include 1) examining a novel target of stimulation that differs from the classical and contemporary approach; 2) use of a generalizable rehabilitation paradigm that is delivered over the long term to generate true estimates of efficacy, adherence, and retention, thence, utility of brain stimulation; 3) use of a method of stimulation that is low cost, safe, feasible and allows estimation of the magnitude of the placebo effect; and 4) characterization of structural, pathologic, neurophysiologic and functional predictors of response. Despite these strengths, we anticipate challenges. The estimated sample size may be limited. Because others have noted significant effects with similar repeated-measures designs with a total of 14 [30] to 24 [8] patients, we anticipate our pilot exploratory study may generate comparable effects. Nevertheless, we do not wish to undermine the significance of large-scale studies. In fact, our current exploratory study is a step in the direction. The resources of our study and its single-center nature, though, are restrictive in addressing efficacy at that level at this time. Combining imaging and stimulation makes enrollment increasingly difficult and adds greater measurement burden. Also, since the intervention can only be delivered in a hospital setting, poor compliance can affect feasibility. To mitigate such threats, a contracted treatment schedule for CIMT is being followed. If a greater and/or accelerated benefit were to be achieved with tDCS, then the utility of abridged CIMT would be established. Our rationale is derived from our work where tDCS paired with contracted vision rehabilitation promoted gains equivalent to a traditional, longer paradigm [32,40,71]. Ultimately, our pilot clinical study carries important implications for future trials; utility of an alternate cortical target would inform future transcranial research and investigation of markers of recovery would inform invasive (intracranial) applications of subcortical/deep brain structures that are in translational stages [72,73].

### Trial status

The trial is actively enrolling.

## Abbreviations

CIMT: Constraint-induced movement therapy; Fc: Functional connectivity; fMRI: Functional magnetic resonance imaging; GCP: Good clinical practice; M1: Primary motor cortex; NIH: National Institutes of Health; PMC: Premotor cortex; rTMS: Repetitive transcranial magnetic stimulation; tDCS: Transcranial direct current stimulation; TMS: Transcranial magnetic stimulation; UEFM: Upper extremity Fugl-Meyer.

## Competing interests

AM has the following conflicts of interest to disclose. None of these are directly pertinent to this research: Intelect medical (advisory board, consultant, shareholder), ATI and cardionomics (shareholder) monteris (consultant).

## Authors’ contributions

All authors made significant intellectual contributions. EP is the initiator and principal investigator. DC has developed data collection and the analysis scheme for TMS. GY created the clinical trial design with EP. AW and CB are trial managers and research coordinators. EB, KS and ML have helped develop the imaging measurement scheme for diffusion tensor imaging, fc and fMRI. SJ has helped develop fMRI analysis and lesion profile for stroke. XW is the trial statistician. AM developed the approach and protocol with EP and edited and helped draft the manuscript. EP created the first draft that was reviewed and edited critically by all other authors, and their revisions were included by EP. All authors read and approved the final manuscript.

## Authors’ information

Study Statistician: Xiao-Feng Wang.
